# The effects of caffeine on olfactory function and mood: an exploratory study

**DOI:** 10.1007/s00213-020-05695-6

**Published:** 2020-10-29

**Authors:** Lorenzo D. Stafford, Kaylee Orgill

**Affiliations:** Department of Psychology, University of Portsmouth, King Henry Building, King Henry I Street, Portsmouth, PO1 2DY UK

**Keywords:** Smell, Adenosine, Coffee, Tea, Odour threshold, Odour identification

## Abstract

Caffeine has been demonstrated to enhance olfactory function in rodents, but to date, the sparse research in humans has not shown any equivalent effects. However, due to the methodological nature of those human studies, a number of questions remain unanswered, which the present study aimed to investigate. Using a double-blind experimental design, participants (*n* = 40) completed baseline mood measures, standardised threshold and identification tests and were then randomly allocated to receive a capsule containing either 100 mg of caffeine or placebo, followed by the same olfactory tests and mood measures. Results revealed that despite a trend toward elevated arousal following caffeine for habitual caffeine consumers, there were no changes in odour function. In contrast, for non-caffeine consumers, caffeine acted to enhance odour (threshold) sensitivity but reduce odour identification. Overall, these findings demonstrate a complex profile of effects of caffeine on odour function and, given the evidence from the wider caffeine literature, it is proposed that the effects of caffeine might be limited to older populations.

## Introduction

Caffeine is contained in a number of common beverages such as tea, coffee and energy drinks and has been consumed for over 2000 years (Barone and Roberts 1984). Caffeine is classified as a psychostimulant and has been extensively researched for its effects over the years, which include increases in arousal and enhanced performance in tasks requiring vigilance (see review Temple et al. 2017). However, there is still debate on the veracity of such effects in those who regularly consume caffeine, i.e. are the observed effects more about reversing the withdrawal symptoms of caffeine (James and Rogers 2005). At doses routinely consumed by humans, the main mechanisms of action are the antagonism of adenosine receptors (Patocka et al. 2019) and of particular interest here are its effects on the adenosine A_2a_ receptors. Research has shown that the stimulatory effects of caffeine are largely achieved via the blockage of adenosine A_2a_ receptors (Svenningsson et al. 1999), and separately, we know that A_2a_ receptors are found in the olfactory bulb (Kaelin-Lang et al. 1999). Evidence for the link between adenosine and odour function was also demonstrated in an elegant rodent experiment where both caffeine and separately an A_2a_ receptor antagonist enhanced olfactory function (Prediger et al. 2005). Since no effects were found for an adenosine A_1_ receptor antagonist, that study suggested that the enhancements observed were due to caffeine’s action as a partial A_2a_ receptor antagonist.

Due to the importance of our sense of smell (Stevenson 2010; Philpott and Boak 2014) and the ineffectiveness of interventions for anosmic/hyposmic individuals (Philpott and Boak 2014; Lill et al. 2006), there is growing interest in the possibility that caffeine might have positive effects on olfaction in humans. This has also been bolstered by one study that found that in humans with first-degree relatives of Parkinson’s disease patients, olfactory function was higher for those who consumed more caffeine (Siderowf et al. 2007). To date, only two studies have tested the effects of caffeine on olfactory function in humans, where one study found that caffeine administration had no effect on a group of hyposmic (impaired sense of smell) individuals (Meusel et al. 2016). The second study tested individuals without any known smell impairments and despite caffeine improving attention (fewer errors), there were no differences on odour function (Han et al. 2020).

Though these two studies suggest that caffeine has no effect on odour function, there are however some aspects that remain unclear. Both of the previous studies used coffee (caffeinated versus decaffeinated) as the method of administering caffeine, which, although having high ecological validity, does also introduce issues in terms of the additional active compounds found in coffee (Arnaud 2011) and hence does not answer the question of whether caffeine alone might influence odour function. It is also likely that there would be expectancy effects from individuals receiving such beverages (both caffeinated/decaffeinated) which may have affected subsequent behaviour and have been found in caffeine research (e.g. Dawkins et al. 2011). Additionally, both of those studies used a relatively modest and similar dose of caffeine, estimated at 65 mg (Meusel et al. 2016) and 72 mg (Han et al. 2020), and it is therefore unclear whether a larger dose might yield any differences in odour function. Finally, neither of the studies measured individual mood and hence it was uncertain whether the stimulant effects of caffeine were present. To answer these questions, the current experiment examined the effects of a dose of caffeine (100 mg) shown to have mood effects (e.g. Smit and Rogers 2000; Stafford and Yeomans 2005) on odour function and mood in a healthy sample. We also took measures of caffeine craving (West and Roderique-Davies 2008) to verify whether individuals who were overnight deprived of caffeine differed in the subsequent caffeine and placebo conditions and how this related to olfaction.

## Method

### Participants

Participants (*n* = 40; age *M* = 19.3, *SD* = 1.9 years, range 18–29; 34 females, 6 males) were university students. Sample size (*N* = 40) was predetermined by a combination of power analysis calculations and previous work in this area. Power analysis (G*Power 3.1) for ANOVA, given *f* = 0.50, power 0.8 and *α* = 0.05, recommends *N* = 34 participants, but we opted for a more cautious *N* = 40. Effect size was based on a previous work testing olfactory function (Stafford and Welbeck 2010; Stafford et al. 2019). Participants were advised not to take part if they had respiratory problems (e.g. asthma): problems in their ability to smell and/or allergies to certain odourants. Additionally, due to the administration of caffeine, we specified that individuals with any known aversions to food additives (aspartame, saccharin, fructose, glucose, sucrose, caffeine, natural food colouring, maltodextrin) should not take part. The study was advertised as ‘Understanding our sense of smell’, and the protocol (see Table 1) was given ethical approval from the University’s Science Faculty Ethics committee (SFEC 2018-095); all participants gave informed consent.

**Table 1:** Timeline representing flow of experiment

Task	Minutes pre/post treatment
POMS (1)	− 30
Odour threshold (1)	− 25
Odour identification (1)	− 10
*Treatment—capsule*	*0*
POMS (2)	30
Odour threshold (2)	35
Odour identification (2)	50
Caffeine craving (QCC)	60
General health form	65
Participant question form	67
Finish	70

*POMS* Profile of Mood States, *QCC* Questionnaire of Caffeine Craving

### Design

The study used a between-subjects design, where individuals were randomly assigned to a caffeine or placebo condition and the main dependent variables were odour sensitivity (threshold), odour identification and mood.

#### Olfactory threshold

The odour used for the threshold test was *n*-butanol (Fisher Scientific, UK) which was diluted in distilled water. The odourant was prepared using fifteen 50-ml amber glass bottles, in 16 dilution steps, starting at 1% (step 1) with each successive step diluted by a factor of two using serial dilution to the lowest (step 16). In addition to the odour containing bottles, for each dilution step, two ‘blank’ bottles (containing dilutant only) were used in the threshold test. Testing commenced by asking participants to smell the bottle with the highest concentration to familiarise themselves with the target odour. They were then presented with the triplet containing the weakest concentration. Following presentation of the last bottle of the triplet (counterbalanced), participants were asked which bottle contained the odour (1, 2 or 3). If the participant answered correctly (and it was the lowest concentration), they were presented with the same triplet again (in a different order) and the task repeated until they made a mistake, which resulted in the triplet containing the next concentration step being presented. Using a single up-down staircase system (as used widely in olfactory research, e.g. (Kobal et al. 1996; Hummel et al. 2007)), this was then repeated until there were seven ‘turning points’, with the mean of the last four points determining the threshold for the individual. Each bottle was held under the participant’s nose (≈ 2 cm) and gently waved between each nostril to ensure optimal inhalation. A blindfold was used by the participants to avoid odour identification. The experimenter wore cotton gloves (Boots, Portsmouth) to reduce any cross contamination of odours.

#### Odour identification

This task was closely modelled on the Sniffin’ sticks identification test (Hummel et al. 2007). In this version, we used fifteen different odourants: lavender (Essential oil, Holland and Barret, 3 drops), glue (PVA, 3 drops), sandalwood (Essential oil, Mia Roma, 3 drops), nutmeg (Tesco, small section), oil (WD40, 1 spray), vanilla extract (Tesco, 3 drops), star anise (Tesco, small section), cinnamon (Schwartz, 1 ml), pear (isoamyl acetate, Fisher Scientific, 1 ml), tea leaves (Tesco, 2 ml), chocolate (Dale Air, 1 ml), thyme (Sainsburys, 2 ml), frankincense (essential oil, Holland and Barret, 3 drops), caraway (Tesco) and oregano (Tesco, 2 ml). For each odourant, the respective amount was placed on a cotton ball (Boots) if a liquid or under the cotton ball; all odourants were then placed in an individual amber glass bottle (50 ml). Participants were presented with one odour at a time and asked to identify which odour they had smelled from a form consisting of four possible odours. They were instructed to make a choice even if they were unsure or did not detect an odour. To minimize practice effects, there were two different versions of the task, which varied in the order the odours were presented and also in the order the odours appeared on the form. We completed piloting of the test to ensure performance was neither at floor or ceiling levels.

#### Profile of Mood States

We used a briefer version composed of the original 72-item Profile of Mood States (POMS) questionnaire (McNair et al. 1971), which composed of 39 items from the original inventory, with the addition of ‘jittery/nervous/shaky’, ‘headache’, ‘hungry’ and ‘calm’, which were included to measure withdrawal and general effects of caffeine. The rationale for using a shorter version was based on the premise that a number of factors were not relevant to caffeine research, i.e. ‘Anger’, ‘Depression’ and ‘Elated’, and was used in previous work (Stafford and Yeomans 2005). Subjects rated the 43 items on a 5-point scale from ‘Not at all’ to ‘Extremely’. From these responses, POMS permits five factors to be extracted: ‘anxiety’, ‘fatigue’, ‘vigour’, ‘confusion’ and ‘friendliness’ and the additional factor of ‘arousal’ (anxiety + vigour) − (fatigue + confusion).

#### Caffeine Craving Questionnaire

The current study used the Questionnaire of Caffeine Craving (QCC; West and Roderique-Davies 2008) which was based on the Questionnaire of Smoking Urges (QSU) (Tiffany and Drobes 1991). The QCC is a 21-item measure, yielding three factors: factor 1 (Desires and intention), factor 2 (General reinforcement) and factor 3 (Negative reinforcement).

#### General Health Questionnaire

This form contained of five questions concerning the frequency of consuming tea, coffee and soft drinks which was used in previous work (Stafford et al. 2010), followed by the frequency of smoking/vaping and alcohol consumption.

#### Caffeine administration

Pre-weighed quantities of caffeine hydrochloride and a white powder used as a placebo (maltodextrin) were stored in small transparent non-gelatine vegetarian capsules (all items supplied by Bulk Powders UK) (size 4) in coded plastic boxes to ensure double-blind testing. The quantity of caffeine/placebo was 100 mg.

### Procedure

Participants were instructed to refrain from consuming any food/drinks that contained the following substances, alcohol, taurine, caffeine, glucose and aspartame, for 12 h before their allocated session. On arrival, participants were asked what they had consumed in the last 12 h and any participants who had consumed any of the listed substances were rescheduled to another session; they then completed baseline measures (POMS, odour threshold, odour identification). They were then given the capsule with a glass of water. This was followed by a rest period (30 min) to allow for the caffeine to be metabolised. Next, they completed the same tasks in the same order; the version for the odour identification task was different to the first presentation. Following the completion of these tasks, they completed the QCC, general health form and were asked two questions: (a) What did they think was the aim of the study? (b) Did they think the capsule they consumed contained caffeine: Y/N? Finally, they were given a full debriefing.

### Data analyses

Preliminary analyses revealed that one participant did not achieve a threshold score, even at the highest concentration and was therefore excluded from further analyses.

Sample characteristics (Table 2) showed that some participants (*n* = 8) were not habitual caffeine consumers, and although this was not a primary aim of the study, we decided to allocate these participants to a Non-consumer group to compare against Consumers. There were an equal number (*n* = 4/4) of participants in the caffeine and placebo conditions, and there were no differences in age in any of the (Condition—Caffeine/Placebo and Group—Consumer/Non-consumer) comparisons. Gender was evenly spread in the Condition/Group combinations (Table 2).

**Table 2:** Mean (SD) participant characteristics by Condition (Caffeine/Placebo)

	Caffeine	Placebo
Age	19.0 ± 1.0	19.6 ± 2.5
Gender (F/M)	18/2	16/4
Number of non-habitual caffeine consumers	4	4
Gender (F/M)	4/0	3/1
Caffeine (mg per day*)	223.1 ±152.2	171.6 ±72.7
Number of smokers	2	2

*Excluding non-consumers

Data for odour threshold, odour identification and mood were calculated as differences from baseline. These data were then analysed using a multivariate ANOVA, with the between-subjects factors of Condition (Caffeine/Placebo) and Caffeine status (Consumer/Non-consumer). Caffeine craving data was also analysed with the same multivariate ANOVA. Preliminary analyses of the data revealed that Box’s Test of Equality of Covariance Matrices was violated, Box’s *M* = 53.25, *F* = 1.99, *p* = .009, which was due to differences in variability in the Non-consumer group, particularly in the Desires and intention factor.

## Results

### Odour threshold

**Fig. 1:**
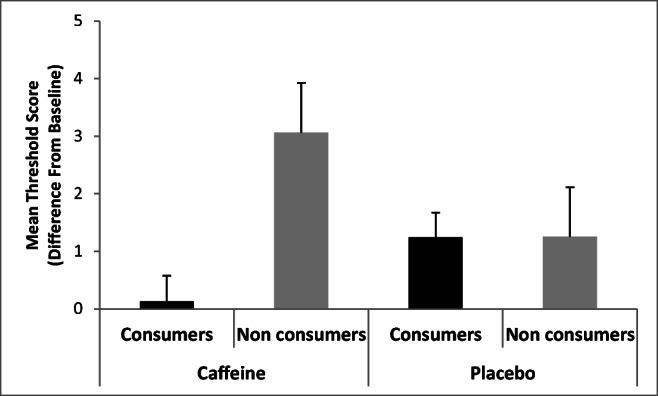
Mean odour threshold by condition (Caffeine/Placebo) and Group (Consumer/Non-consumer)

Analyses revealed no significant main effect of Condition, *F*(1,35) = 0.27, ns, with sensitivity increasing across both the caffeine and placebo conditions (Table 2). There was, however, a main effect of Group, *F*(1,35) = 4.63, *p* = .038, *η*^2^ = .12, qualified by a Condition × Group interaction, *F*(1,35) = 4.53, *p* = .04, *η*^2^ = .12. Further analyses revealed that there were no differences between caffeine consumers and non-consumers following placebo (*p* = .99), but in contrast for caffeine, the non-consumers sensitivity was higher than the consumers (*p* = .016) (Fig. 1).

#### Odour identification

**Fig. 2:**
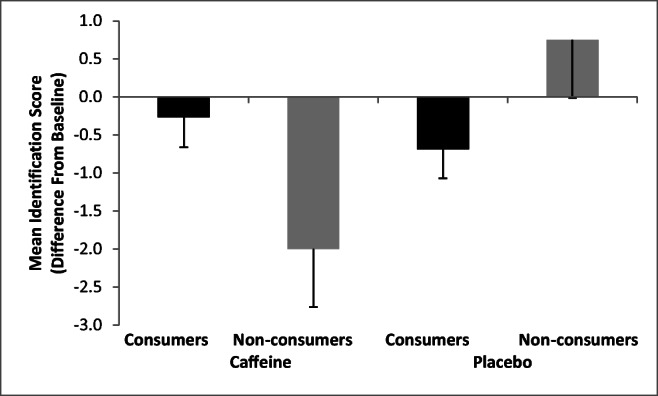
Mean odour identification by Condition (Caffeine/Placebo) and Group (Consumer/Non-consumer)

For odour identification, the main effect of Condition was not significant *F*(1,35) = 3.72, *p* = .06, though interestingly, there was a trend toward poorer performance following caffeine (*M* = − 1.13, SD = 1.76) compared with placebo (*M* = 0.03, SD = 1.34). The main effect of Group was not significant, *F*(1,35) = 0.06, ns, but there was a significant Condition × Group interaction, *F*(1,35) = 6.90, *p* = .01, *η*^2^ = .17. Further analyses demonstrated this to be due to no differences of Condition for caffeine consumers (*p* = .39), whereas for non-consumers, caffeine led to poorer performance compared with placebo (*p* = .059) (Fig. 2; Table 3).

**Table 3:** Mean (SD) odour function, cravings and mood by Condition (Caffeine/Placebo) and Group (Consumer/Non-consumer)

	Caffeine		Placebo	
	Consumers	Non-consumers	Consumers	Non-consumers
Threshold score*	0.13 ± 1.7	3.06 ± 2.75	1.24 ± 1.57	1.25 ± 1.10
Identification	− 0.27 ± 1.53	− 2.0 ± 2.0	− 0.69 ± 1.45	0.75 ± 1.25
Tense/anxiety	− 0.67 ± 2.79	0.00 ± 3.36	− 1.94 ± 2.67	2.23 ± 3.09
Arousal	2.67 ± 6.98	2.75 ± 5.38	− 1.31 ± 5.72	4.00 ± 2.16
Craving:				
Desires and intention	2.68 ± 1.43	1.21 ± 0.26	2.21 ± 1.03	1.11 ± 0.17
Gen reinforcement	4.71 ± 1.17	3.35 ± 1.22	4.70 ± 1.17	3.0 ± 1.34
Neg reinforcement	3.92 ± 1.55	1.80 ± 0.63	2.84 ± 1.23	3.20 ± 1.72

*Higher numbers represent greater odour sensitivity

#### Mood

The analyses revealed no main effects of Condition on any of the mood measures (all *F*’s < 1.3); however, when analysed separately for each group, differences did emerge. For Consumers only, arousal ratings increased for those receiving caffeine but declined for those in the placebo group, *F*(1,29) = 3.03, *p* = .09, *η*^2^ = .09. Tense/anxiety ratings declined more sharply for placebo compared with caffeine, *F*(1,29) = 3.64, *p* = .06, *η*^2^ = .11 (Table 3). The mood data therefore show non-significant trends for the stimulant effects of caffeine in caffeine consumers but not in non-consumers.

#### Caffeine craving

Craving measures showed a main effect of Group for Desires and intention, *F*(1,35) = 8.25, *p* = .007, *η*^2^ = .19 and General reinforcement, *F*(1,35) = 9.71, *p* = .004, *η*^2^ = .22 where, unsurprisingly, craving was higher for consumers versus non-consumers (Table 3). There were no effects for Condition (all *F*’s < 1), but there was a significant Condition × Group interaction for Negative reinforcement, *F*(1,35) = 5.16, *p* = .02, *η*^2^ = .13, with further analyses showing that for Consumers only, ratings were higher following caffeine compared with placebo.

## Discussion

The study found that, overall, there were no significant effects of caffeine on odour threshold or identification, which is consistent with previous work (Meusel et al. 2016; Han et al. 2020).

Both of those studies utilized coffee as the vehicle for delivering caffeine and since coffee also contains other active compounds (Arnaud 2011); the effects of caffeine alone on odour function were unknown. By using pure caffeine contained in capsules, the current study was able to overcome that limitation and additionally examine the effects of using a larger dose of caffeine. The findings here suggest that neither coffee containing caffeine nor caffeine alone have any overall effect on odour function. Interestingly, however, there were differences between habitual caffeine consumers and non-consumers which were not investigated in the earlier work (Meusel et al. 2016; Han et al. 2020). For non-consumers only, caffeine had divergent effects, leading to higher odour sensitivity (threshold test) compared with consumers but, in contrast, reduced odour identification. In trying to account for these differences, it could be that the stimulatory effects of caffeine were particularly beneficial for the threshold test, being a task longer in duration and possibly monotonous to some individuals, whereas the same arousing effects were not beneficial in the identification test, being a shorter task, demanding higher order cognitive function. This theory would also seem to fit the pattern that caffeine has a more reliable effect on attention and vigilance rather than memory and more demanding cognitive tasks (Stafford et al. 2007).

The reason that consumers’ odour function did not follow the same pattern could be explained in that they would be less sensitive to the effects of caffeine, and therefore, following a period of caffeine abstinence, consumer ingestion of caffeine may have simply reversed caffeine withdrawal (James and Rogers 2005). Nevertheless, it was curious that the best evidence of any changes in mood was for the consumers only, in terms of tense and arousal, though only reaching significance in the former. Such differences do however link to the wider caffeine literature which has shown differences in the effects of caffeine in consumers and non-consumers. For instance, one study found that caffeine benefitted performance (vigilance) more in non-consumers versus consumers but in contrast for mood, consumers derived more benefit from caffeine than non-consumers (Haskell et al. 2005). Drawing on these separate areas, it could be, therefore, that any caffeine-induced alterations in odour function are not dependent on observable changes in mood.

The absence of caffeine effects on olfaction needs to be considered in the wider context. Previous human work that suggested positive effects was based on first-degree relatives of Parkinson’s disease patients, where increasing lifetime estimates of caffeine intake were associated with higher olfactory (UPSIT) function (Siderowf et al. 2007). One obvious difference between that work and the study here is that no caffeine was administered in that study but rather relied on individuals’ account of routine caffeine intake. It is also worth noting that the participants in that study were all over 50 years of age and hence substantially older than the study here (*M* = 19 years) and the previous study in normosmics (27 years; Han et al. 2020). This fact could be important in that the enhancing effects of caffeine on olfaction in animal work were based on older (12 and 18 months) rodents (Prediger et al. 2005) and therefore suggest that the beneficial effects of caffeine on odour function might be restricted to older humans. The suggestion from that work was that the age-related increase in adenosine A_2a_ receptors may play a role in declining odour function, which can be temporarily reversed by caffeine’s antagonism of those adenosine A_2a_ receptors. One of the consequences of this blockade is the increasing transmission of a range of neurotransmitters including dopamine, noradrenaline, and glutamate in brain areas related to cognitive function (see review, Patocka et al. 2019). In summary, in accordance with the wider research on the effects of caffeine in ageing (e.g. Van Gelder et al. 2007), it could be that caffeine may influence olfactory function but that this is mainly limited to older individuals.

In terms of the study limitations, it is important to acknowledge that whilst the overall sample size used in this study was adequate in terms of the pre study power calculations, the number of non-consumers in this study (*n* = 8) was rather small and therefore the findings relating to that group need to be treated as preliminary. It is also worth reflecting that although the dose of caffeine used here was larger than previous work on odour function (Meusel et al. 2016; Han et al. 2020), it is uncertain whether using a larger dose may lead to different effects, which given caffeine’s rather inconsistent effects (Stafford 2004; Stafford et al. 2007), would be worth examination. Finally, the odour ‘identification’ test used in this study was a custom-built test, used for the first time, and even though modelled closely on the Sniffin’ sticks identification test (Kobal et al. 1996), it was not a validated test (see considerations, Hsieh et al. 2017). Nevertheless, we did complete pilot testing before the study to ensure that the test was sensitive to detect effects and performance was not at ‘floor’ or ‘ceiling’ levels.

In conclusion, we found no overall effect of caffeine on odour function, but in evidence that for non-consumers only, caffeine had beneficial effects on odour threshold but impaired odour identification.

## References

[CR1] Arnaud MJ (2011) Pharmacokinetics and metabolism of natural methylxanthines in animal and man. Handbook of Experimental Pharmacology 33–91. 10.1007/978-3-642-13443-2_3 10.1007/978-3-642-13443-2_3 20859793

[CR2] Barone JJ, Roberts H, Dews PB (1984). Human consumption of caffeine. Caffeine: perspectives from recent research.

[CR3] Dawkins L, Shahzad FZ, Ahmed SS, Edmonds CJ (2011). Expectation of having consumed caffeine can improve performance and mood. Appetite.

[CR4] Han K, Lee J, Choi BY, Jeong H, Cho JH, Kim JK (2020). Does improved attention induced by caffeine intake affect olfactory function?. Clin Exp Otorhinolaryngol.

[CR5] Haskell CF, Kennedy DO, Wesnes KA, Scholey AB (2005). Cognitive and mood improvements of caffeine in habitual consumers and habitual non-consumers of caffeine. Psychopharmacology.

[CR6] Hsieh JW, Keller A, Wong M, Jiang RS, Vosshall LB (2017). SMELL-S and SMELL-R: olfactory tests not influenced by odor-specific insensitivity or prior olfactory experience. Proc Natl Acad Sci.

[CR7] Hummel T, Kobal G, Gudziol H, Mackay-Sim A (2007). Normative data for the “Sniffin’ sticks” including tests of odor identification, odor discrimination, and olfactory thresholds: an upgrade based on a group of more than 3,000 subjects. Eur Arch Otorhinolaryngol.

[CR8] James JE, Rogers PJ (2005). Effects of caffeine on performance and mood: withdrawal reversal is the most plausible explanation. Psychopharmacology.

[CR9] Kaelin-Lang A, Lauterburg T, Burgunder JM (1999). Expression of adenosine A2a receptors gene in the olfactory bulb and spinal cord of rat and mouse. Neurosci Lett.

[CR10] Kobal G, Hummel TH, Sekinger B, Barz S, Roscher S, Wolf S (1996). “Sniffin’ sticks”: screening of olfactory performance. Rhinology.

[CR11] Lill K, Reden J, Müller A, Zahnert T, Hummel T (2006) Olfactory function in patients with post-infectious and post-traumatic smell disorders before and after treatment with vitamin A: a double-blind, placebo-controlled, randomized clincial trial. Chem Senses 31:A33 10.1002/lary.23405 22752966

[CR12] McNair DM, Lorr M, Droppleman L (1971). *Manual for the profile of mood states, educational and industrial testing service*.

[CR13] Meusel T, Albinus J, Welge-Luessen A, Hähner A, Hummel T (2016). Short-term effect of caffeine on olfactory function in hyposmic patients. Eur Arch Otorhinolaryngol.

[CR14] Patocka J, Navratilova Z, Krejcar O, Kuca K (2019). Coffee, caffeine and cognition: a benefit or disadvantage?. Lett Drug Des Discov.

[CR15] Philpott CM, Boak D (2014). The impact of olfactory disorders in the United Kingdom. Chem Senses.

[CR16] Prediger RD, Batista LC, Takahashi RN (2005). Caffeine reverses age-related deficits in olfactory discrimination and social recognition memory in rats: involvement of adenosine A1 and A2A receptors. Neurobiol Aging.

[CR17] Siderowf A, Jennings D, Connolly J, Doty RL, Marek K, Stern MB (2007). Risk factors for Parkinson’s disease and impaired olfaction in relatives of patients with Parkinson’s disease. Mov Disord.

[CR18] Smit H, Rogers PJ (2000). Effects of low doses of caffeine on cognitive performance, mood and thirst in low and higher caffeine consumers. Psychopharmacology.

[CR19] Stafford LD (2004) What makes caffeine reinforcing?: salient factors and cognitive mechanisms (Doctoral dissertation, University of Sussex)

[CR20] Stafford LD, Welbeck K (2010). High hunger state increases olfactory sensitivity to neutral but not food odors. Chem Senses.

[CR21] Stafford LD, Yeomans MR (2005). Caffeine deprivation state modulates coffee consumption but not attentional bias for caffeine-related stimuli. Behav Pharmacol.

[CR22] Stafford LD, Rusted J, Yeomans MR (2007). Caffeine, mood and performance: a selective review. Caffeine and activation theory: effects on health and behavior.

[CR23] Stafford LD, Wright C, Yeomans MR (2010). The drink remains the same: Implicit positive associations in high but not moderate or non-caffeine users. Psychol Addict Behav.

[CR24] Stafford LD, Damant K, Ashurst S, Parker MO (2019) Higher olfactory sensitivity to coffee odour in habitual caffeine users. Exp Clin Psychopharmacol 10.1037/pha0000293 31070427

[CR25] Stevenson RJ (2010). An initial evaluation of the functions of human olfaction. Chem Senses.

[CR26] Svenningsson P, Nomikos GG, Fredholm BB (1999). The stimulatory action and the development of tolerance to caffeine is associated with alterations in gene expression in specific brain regions. J Neurosci.

[CR27] Temple JL, Bernard C, Lipshultz SE, Czachor JD, Westphal JA, Mestre MA (2017). The safety of ingested caffeine: a comprehensive review. Front Psychiatry.

[CR28] Tiffany ST, Drobes DJ (1991). The development and initial validation of a questionnaire on smoking urges. Br J Addict.

[CR29] Van Gelder BM, Buijsse B, Tijhuis M, Kalmijn S, Giampaoli S, Nissinen A, Kromhout D (2007). Coffee consumption is inversely associated with cognitive decline in elderly European men: the FINE Study. Eur J Clin Nutr.

[CR30] West O, Roderique-Davies G (2008). Development and initial validation of a caffeine craving questionnaire. J Psychopharmacol.

